# Balancing evidence-informed and user-responsive design: Experience with human-centered design to generate layered economic empowerment and SRH programming in Tanzania, Ethiopia, and Nigeria

**DOI:** 10.12688/gatesopenres.14724.1

**Published:** 2023-07-04

**Authors:** Meghan Cutherell, Mary Phillips, Carrie Ellett, Emnet Woubishet, Joy Otsanya Ede, Akinjide Adesina, Arnold Kabahaula, Alex Nana-Sinkam, Abednego Musau, Katherine Nichol

**Affiliations:** 1 Population Services International, Washington, District of Columbia, 20036, USA; 2 BRAC International, Nairobi, Kenya; 3 Population Services International Ethiopia, Addis Ababa, Ethiopia; 4 Society for Family Health Nigeria, Abuja, Federal Capital Territory, Nigeria; 5 Population Services International Tanzania, Dar es Salaam, Tanzania; 6 IDEO.org, Nairobi, Kenya; 7 Kore Global, Vancouver, Canada

**Keywords:** Human-centered Design (HCD), economic empowerment, sexual and reproductive health (SRH), adolescents, user-centered, evidence-based, gender-responsive, Adolescents 360 (A360)

## Abstract

In 2021, the Adolescents 360 (A360) project pursued a human-centered design (HCD) process to layer complementary economic empowerment components on top of its existing sexual and reproductive health (SRH) interventions targeting adolescent girls aged 15 to 19. Given the volume of evidence informing successful approaches for improving economic and empowerment outcomes for adolescents, we pursued an intentionally evidence-informed and gender-intentional design process, while trying to also respond directly to user insights. In this open letter, we share how we utilized and validated the evidence-base while applying the core tenets of HCD (empathy and user insights) to design holistic, layered programming for girls. We describe three overarching categories which depict how we used the existing evidence and new user insights to strengthen our design process. Often the evidence base allowed us to expedite finding a solution that worked for our users. However, at times there was a disconnect between what we knew worked in the evidence base and what our users said they wanted. New insights also allowed us to build a greater understanding of our users’ lived experiences where there were existing evidence gaps. We were aided by the engagement of a technical partner, BRAC, who synthesized evidence for our design teams and functioned as an ‘on demand’ support mechanism as questions and challenges arose. Yet, the volume of information to absorb almost guaranteed that we would miss out on the opportunity to apply certain evidence-based practices. We encourage researchers to consider how to make evidence more easily digestible to practitioners and for the whole community of practice to work together to identify what questions need to be asked to effectively operationalize evidence in a local context.

## Disclaimer

The views expressed in this article are those of the authors. Publication in Gates Open Research does not imply endorsement by the Gates Foundation.

## Introduction

Adolescent girls represent 16% of all women of reproductive age (15–49) ^1^. Adolescence is a critical moment for intervention, as it is a period that establishes future behaviors and patterns ^2^ and an important window before discriminatory gender norms are fully entrenched ^3^. Adolescents require age-appropriate information, opportunities to develop key life skills, and essential health services in a safe and supportive environment to grow, develop, and pursue healthy futures. Increasing access to tailored, adolescent-focused services and expanding opportunities for adolescents to engage in the design and delivery of interventions related to their own health are critical components of a rights-based approach to adolescent sexual and reproductive health (ASRH) programming ^4^. Yet, the specific needs of adolescent girls are often neglected or underemphasized in global, regional, and national-level priorities.

As girls transition into young women, they face many barriers including school dropout, child marriage, early pregnancy, physical and mental health problems, and gender-based violence ^5^. Lack of sexual and reproductive health (SRH) knowledge and access to comprehensive SRH services, and cultural norms that promote early childbearing contribute to high rates of unplanned pregnancy among adolescents ^6^. Adolescents aged 15–19 have a low contraceptive prevalence rate (CPR), estimated to be around 10.2% and have double the unmet need of all women of reproductive age (43% vs. 24%) ^1^. Simultaneously, discriminatory gender norms and patriarchal structures limit adolescent girls’ ability to achieve economic well-being. These barriers are built-in from an early age and constrain the extent to which girls can gain the skills and experiences necessary to pursue improved economic outcomes and economic autonomy. As adults, women are less likely to participate in the paid labor force compared to men and are more likely to be unemployed and over-represented in informal and vulnerable employment sectors ^7^.

There is a growing evidence base demonstrating adolescent girls’ need for a holistic package of services that can offer reproductive health information and services, build their agency, and establish a positive enabling environment for them to pursue their life goals ^8^. There is increasing interest from the broad community of practice (donors, implementers, and local stakeholders alike) in addressing adolescent girls’ needs through programming that combines SRH with age-appropriate economic empowerment. For adult women, evidence suggests that reproductive health improvements can lead to improvements in economic empowerment ^9– 11^. Yet, for adolescents, the evidence on the interplay between economic empowerment and SRH is more complex. There is some information, albeit limited, on successful programmatic approaches that integrate components of SRH into economic programming for adolescents ^12, 13^. Some evidence also shows that interventions which have sought to prevent early and forced marriage, as well as early childbearing, have had significant impact on the economic empowerment of adolescent girls and young women (AGYW) ^14^. Yet other research demonstrates diluted effects when combining SRH and economic empowerment components in programming for adolescent girls ^15^. Overall, there is a growing body of evidence on the types of interventions which demonstrate significant and meaningful impact on adolescent girls’ economic empowerment ^12^.

### Adolescents 360 Amplify

In 2016, Population Services International (PSI) and its consortium of partners launched Adolescents 360 (A360) with funding from the Bill & Melinda Gates Foundation (BMGF) and the Children’s Investment Fund Foundation (CIFF). A360 was a 4.5-year project working directly with young people to design and deliver interventions that increase demand for, and voluntary uptake of, modern contraception among adolescent girls aged 15 to 19 years. A360 designed and implemented four interventions across three countries – *Smart Start* in Ethiopia, *Kuwa Mjanja* in Tanzania, *Matasa Matan Arewa (MMA)* in northern Nigeria, and *9ja Girls* in southern Nigeria ^16– 21^.

In late 2020, A360 received funding to continue implementation for an additional five years under a follow-on project, A360 Amplify. The strategic priorities for this follow-on include 1) adaptation to strengthen our SRH interventions and to create proof of concept for holistic user-centered intervention components, 2) pursuit of sustainability for our ASRH interventions through institutionalization and scale within government systems, and 3) contribution to filling gaps in the local, regional, and global evidence base through high-quality research and learning activities.

Our user insights indicate that resonance of our interventions in the initial project investment was dependent on offering more than contraceptive counseling and services. By including a life and/or vocational skills and livelihoods demonstration or training (VSLT) component in three out of four interventions (Kuwa Mjanja, MMA, and 9ja Girls), the project responded to what girls said they needed to secure a stable future for themselves. These components made the program feel relevant to girls, helped us to establish the relevance of contraception for girls’ lives, and fostered the approval of girls’ key influencers. However, girls had high expectations for these skills components and for some the reality fell short of their expectations, with girls stating that they needed the opportunity to learn more diverse, marketable skills and to receive additional support to apply these skills for improved income generation.

*“The mentor came to our compound to invite us, she told us that we will be learning about how to take care of our family, about nutrition, FP but what got me interested was that she said at the end, we will learn a skill.…Hearing that … I became so interested.”* – Girl, Nasarawa State; A360 Process Evaluation Nigeria, 2019, Itad ^22^
*“They should be teaching us a variety of skills at a time because parents do stop their girls from participating once they discovered that they are only teaching them just one thing for a long time.”* – Girl, Ogun State; A360 Participatory Action Research Nigeria, 2019, Itad ^23^

This learning clearly demonstrated the value of strengthening these program components to provide a more substantive economic empowerment offering to girls. As a result, in 2020 and 2021, we pursued a human-centered design (HCD) process to layer economic empowerment components onto our existing SRH interventions across all four program geographies. Given the volume of evidence informing successful approaches for improving economic outcomes for adolescents and increasing their empowerment more broadly, we pursued an intentionally evidence-informed and gender-intentional design process, while trying to also respond directly to user insights, maintaining our ‘girl-centered’ approach. In this open letter, we describe our experience cultivating this balance – how we utilized and validated the evidence-base while applying the core tenets of HCD (empathy and user insights) to design holistic, layered programming for girls.

## Design process

Across all geographies, the objective of the design process was to adapt the existing SRH interventions to offer participating girls the opportunity to further identify and progress towards their economic goals. As part of our intentional pursuit of gender transformative programming, we aimed to design age-appropriate program adaptations that not only supported girls to gain additional income, savings, and/or assets but did so in a way that built their self-efficacy and economic autonomy. Given the growing evidence around multi-component approaches that pursue both SRH and economic outcomes, we also wanted to understand how, if at all, these more intensive and intentional economic empowerment components would influence contraceptive uptake and continuation among participating adolescent girls.

The HCD timeline and process was similar across geographies, with some notable differences. We followed the same three overarching design phases as we pursued in our formative design phase for A360’s SRH interventions in 2016 – insight gathering, rough prototyping, and live prototyping. Within each of these phases, potential program participants and other key stakeholders who might interact with the final intervention – such as mothers or husbands – were invited to participate in the testing and give feedback on the evolving concepts. To ensure we included youth perspectives and to help “bridge” the gap between adult researchers and adolescent girl participants, 18–24-year-olds were hired and trained as Youth Innovation Officers (Nigeria / Tanzania) or Young Designers (Ethiopia). These young people participated as full members of the design teams, leading research conversations and prototype testing in the field.

 We partnered with IDEO.org as our HCD technical assistance partner. The COVID-19 pandemic limited in-person engagement and as a result IDEO.org provided design strategy and facilitation remotely, guiding the design and implementation teams in Nigeria, Tanzania and Ethiopia who drove the design process in country. In Ethiopia, our design work began in August 2020 compared to January 2021 for the remaining geographies as the result of a complementary investment from *Maverick Collective* philanthropists. However, in Ethiopia we also had a more intentionally limited engagement from IDEO.org and as a result our design process converged with that of Nigeria and Tanzania in July 2021 during live prototyping. Across all design phases, we engaged 679 adolescent girls (169 in Ethiopia, 96 in southern Nigeria, 156 in northern Nigeria, and 258 in Tanzania) along with 551 other stakeholders (88 in Ethiopia, 149 in southern Nigeria, 196 in northern Nigeria, and 118 in Tanzania) ( Table 1). Quotes from these engagements are provided in this Open Letter to substantiate our learning and experience. The raw data set containing these quotes is currently unpublished, but a synthesis of the data from this design process will be made publicly available at the end of 2023.

**Table 1.** Economic empowerment design phases.

Phase	Who we interacted with	What we did and the expected outcome
**Insight Gathering / ** **Design Research**	**Ethiopia:** • 24 adolescent girls • 19 husbands of adolescent girls • 10 parents-in-law • 6 community leaders • 4 teachers • 2 business owners • 16 local economic empowerment experts	We used qualitative data collection methods to understand participant and community perspectives on girls’ engagement in economic activities. The intent was to surface barriers to girls’ economic participation and empowerment and opportunities to support girls to pursue improved economic outcomes and autonomy. Qualitative methods utilized included: • **Individual interviews** • **Focus group discussions** • **Key informant interviews** • **Role play** • **Vox prop:** Design teams provide participants with a prompt or short story and ask them to respond to that story in a way which conveys their own experiences, thoughts, and beliefs. • **Card sort:** Participants are presented with a set of cards which have different concepts, themes, persons, or topics and are asked to sort these cards based on a prompt from the design team.
	**Southern Nigeria:** • 29 adolescent girls (mostly unmarried) • 20 adolescent boys and older girls • 10 mothers of unmarried adolescent girls • 11 religious and community leaders • 4 female entrepreneurs • 8 government and local organization representatives	
	**Northern Nigeria:** • 30 married adolescent girls • 10 husbands of adolescent girls • 20 mothers-in-law or co-wives of adolescent girls • 10 religious and community leaders • 5 female entrepreneurs • 8 government and local organization representatives	
	**Tanzania:** • 59 adolescent girls (mostly unmarried) • 34 government representatives	
**Rough Prototyping** ***(Including Ideation & *** ***Building of Prototypes)***	**Ethiopia:** • 115 married adolescent girls • 4 husbands of married adolescent girls	In this phase, we used findings from the insight gathering phase to develop hypotheses about how to solve the key challenges and take advantage of opportunities identified. To do this, we built prototypes – models and tangible concepts that people could interact with and respond to. As we received feedback, teams synthesized findings to make iterations to concepts and increase fidelity, or the specificity and resonance of the concepts. • In Nigeria, with hands-on support from IDEO. org the design team moved rapidly, completing this phase in approximately two months. • In Ethiopia, where IDEO.org engaged just for specific moments to help the team build concepts and where there was more synthesis time between field test, this process lasted approximately five months.
	**Southern Nigeria:** • 30 adolescent girls • 16 adolescent boys • 18 community and religious leaders • 8 female entrepreneurs • 1 government representative	
	**Northern Nigeria:** • 30 married adolescent girls • 16 adolescent boys • 18 community and religious leaders • 8 female entrepreneurs • 6 government representatives	
	**Tanzania:** • 70 adolescent girls • 34 other stakeholders (disaggregation of other stakeholders not available)	
**Live Prototyping**	**Ethiopia:** • 30 married adolescent girls • 27 husbands of married adolescent girls	During this phase, we wanted to test the feasibility and scalability of the draft solutions by bringing together different elements from the earlier phases of prototyping into a single ‘test’ that could be experienced by end users as a service or product. Through this test, we learned how different elements of the designed solution worked together as well as learned what resources would be needed to implement the solution in a real- world setting.
	**Southern Nigeria:** • 37 adolescent girls • 49 mothers of adolescent girls • 2 female entrepreneurs • 2 community and religious leaders	
	**Northern Nigeria:** • 96 married adolescent girls • 91 husbands of married adolescent girls • 2 female entrepreneurs • 2 government and organization representatives	
	**Tanzania:** • 82 adolescent girls • 29 parents of adolescent girls • 1 male partner of an adolescent girl • 10 government and organization representatives • 10 community members	

In acknowledgment that economic empowerment programming is not one of PSI’s core technical competencies, throughout the design process, A360 collaborated with BRAC as a conduit to the global evidence base around girls’ economic empowerment. Their technical assistance role, beginning in 2021, included reviews of all design materials, rapid literature summaries, and development of skills or competency-based curriculum, drawing from their evidence-based Empowerment and Livelihoods for Adolescents (ELA) Model ^24^. Their participation in the HCD process was crucial, sharing best practices and evidence that responded to identified needs of adolescents and compiling literature to help guide inquiries that arose in the design process. As the design process evolved, the need for additions to existing A360 curricula content and follow-on support for economic empowerment outcomes became evident. BRAC then engaged in integrating selected ELA content into the A360 curriculum and training staff on its usage. BRAC continued to provide support to our teams as the pilot launched in 2022. 

Kore Global provided technical assistance to pursue a gender-intentional design. With IDEO.org they co-developed a gender in design toolkit to guide the process and ensure the resulting program designs were gender responsive.

## Final intervention designs

The result of the design process is interventions that are informed by the evidence and directly responsive to our users’ lived experiences. While each program aims to achieve the same outcomes, namely increases in girls’ income, savings, and assets, and improved economic autonomy, the specific pathways differ by geography. All programs begin with their respective SRH intervention. Girls are then offered the chance to continue to participate in a more intensive economic empowerment program.

**Figure 1.**
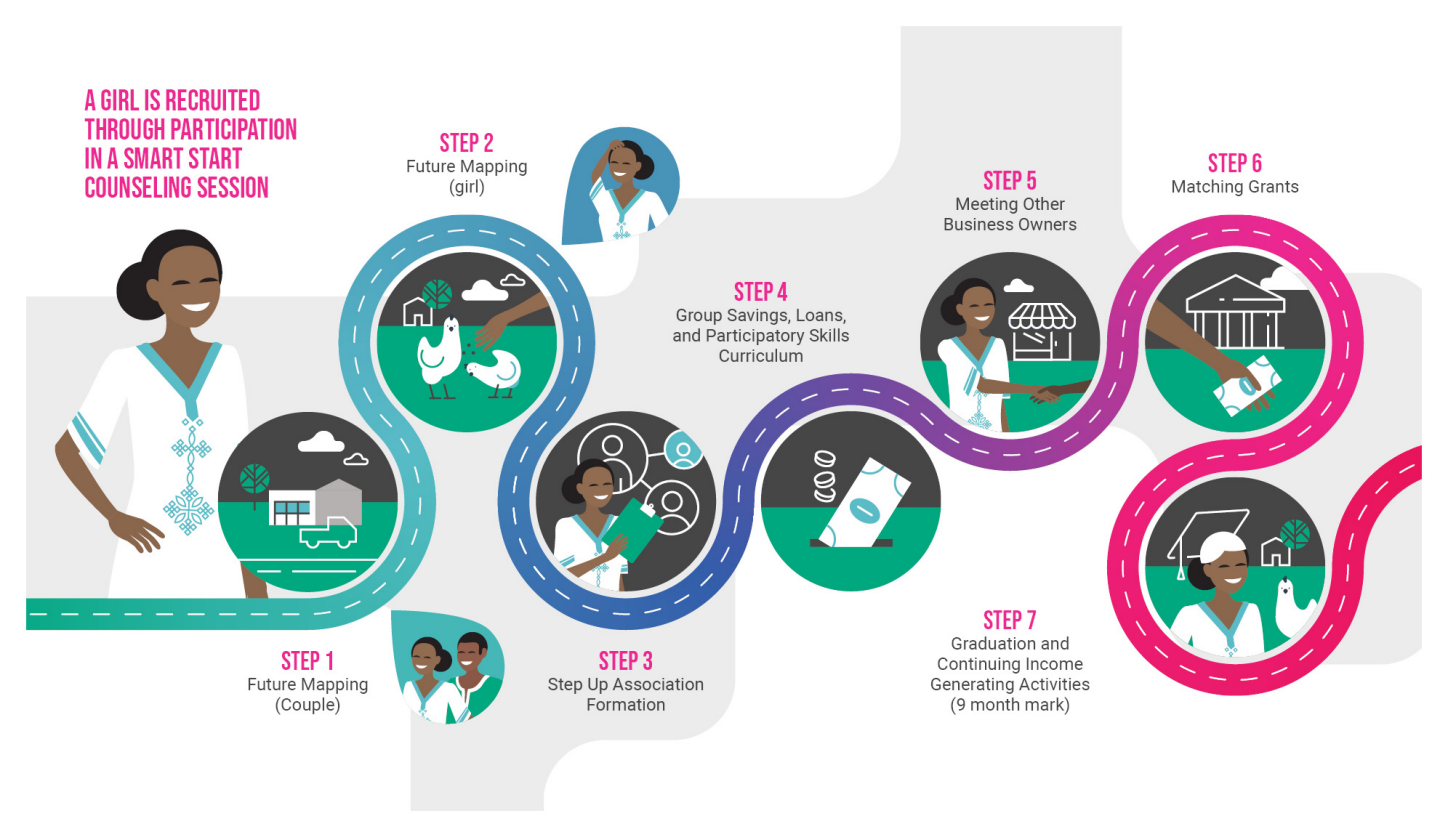
A visual overview of Smart Steps, the economic empowerment adaptation in Ethiopia.

**Figure 2.**
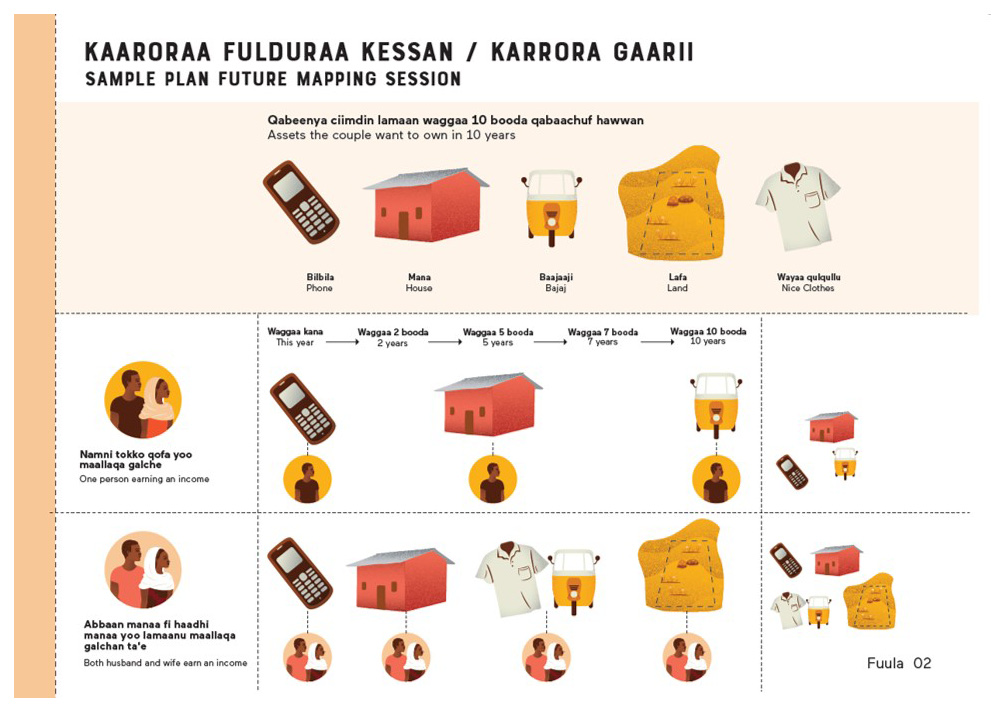
Sample asset, Smart Steps joint future mapping session template.

In Ethiopia, girls are brought into the economic empowerment program, **Smart Steps** ( Figure 1), through a Smart Start counseling session which focuses on how delaying and spacing pregnancies can support girls and couples to pursue financial stability ^19^. If they wish to participate in the expanded economic empowerment program, they are invited to a ‘ **future mapping’ session**, first jointly with their husbands and then individually. During these sessions girls are supported to identify their medium- and long-term goals and what activities they can take to achieve those goals. Girls’ husbands are supported to understand the value of girls’ economic and market participation in accelerating progress towards achieving their joint goals as a couple ( Figure 2). Girls are then gathered into an adapted version of Village Savings and Loan Associations (VSLA), called a **‘Step Up Association**.’ These associations are for married adolescent girls only and self-governed by participants, with support from a program-hired mentor. Each week, the associations meet and provide a platform for girls to collect savings from each member. In our adapted program, girls also go through a **participatory curriculum specifically designed for low-literacy audiences to teach essential soft-skills and business skills.** After four or five weeks of meetings, girls can choose to access loans from the pooled savings to fund investments in existing businesses or to begin new income-generation activities. Groups are eligible for a **matching grant** after the first 16 weeks if: a) all members achieve 80% attendance in the initial period; b) the group has saved a minimum amount; and c) they have complied with their constitution and maintained proper bookkeeping. If the group meets these criteria, they are eligible to have their total savings matched at 150%. This money is transferred to the group as a whole and is then available as part of the loan pool.

**Figure 3.**
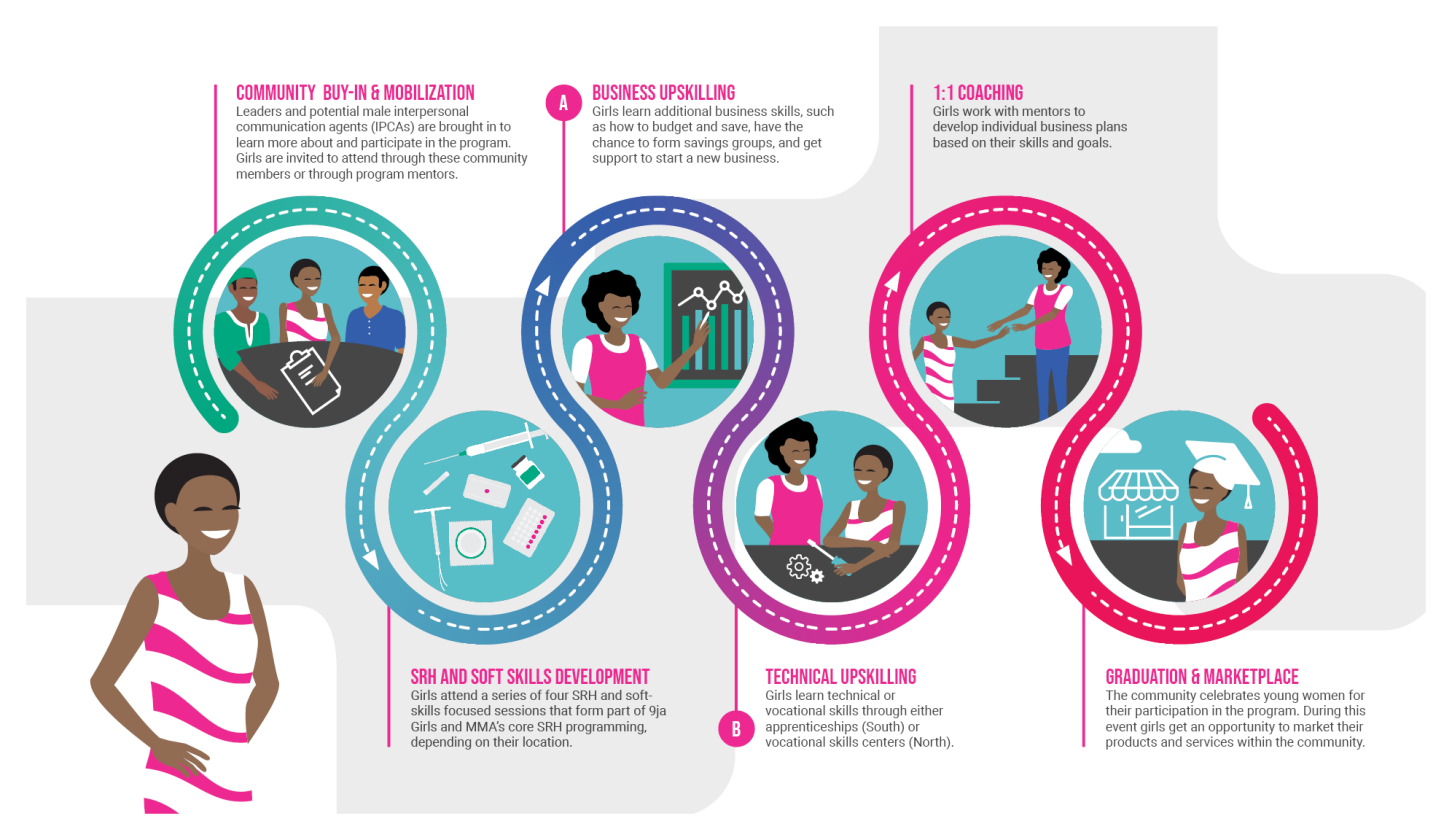
A visual overview of 9ja Girls+ and MMA+, the economic empowerment adaptations in Nigeria.

**Figure 4.**
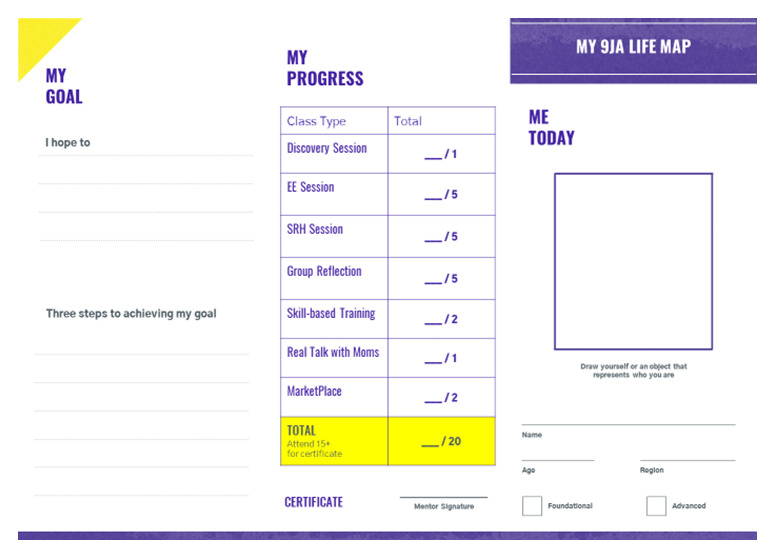
Sample asset, 9ja Girls + life map.

In both northern and southern Nigeria, the heart of the economic empowerment programs, **MMA+** and **9ja Girls+** respectively, is a scaffolded package of soft, business, and vocational skills training combined with mentorship and coaching ( Figure 3 and Figure 4). Communities are oriented to the aims of the joint SRH and economic empowerment program and girls are mobilized, either through referral from their key influencers (primarily parents for unmarried girls and husbands for married girls) or directly by program mentors. They are invited to attend an initial set of **four curriculum sessions, each lasting 90 minutes, that focus on developing health-related knowledge and core soft skills** such as decision-making and communications. After completing these initial sessions, girls are invited to move into a secondary curriculum that provides them with a **combination of business and technical or vocational skills.** After the secondary curriculum, girls can elect to learn one to two vocational skills through either a vocational training center (northern Nigeria) or apprenticeships (southern Nigeria). Following the skills trainings, girls are then given **mentorship support** to develop and execute a business plan. Their participation culminates in a large, public graduation that doubles as a marketplace for girls to display the products and services from their newly gained skills. This is a chance for the community to show public support for the program and for girls to show to their husbands or parents the value of what they have learned through their participation. The program models in southern and northern Nigeria, although serving different girls, are overall very similar.

**Figure 5.**
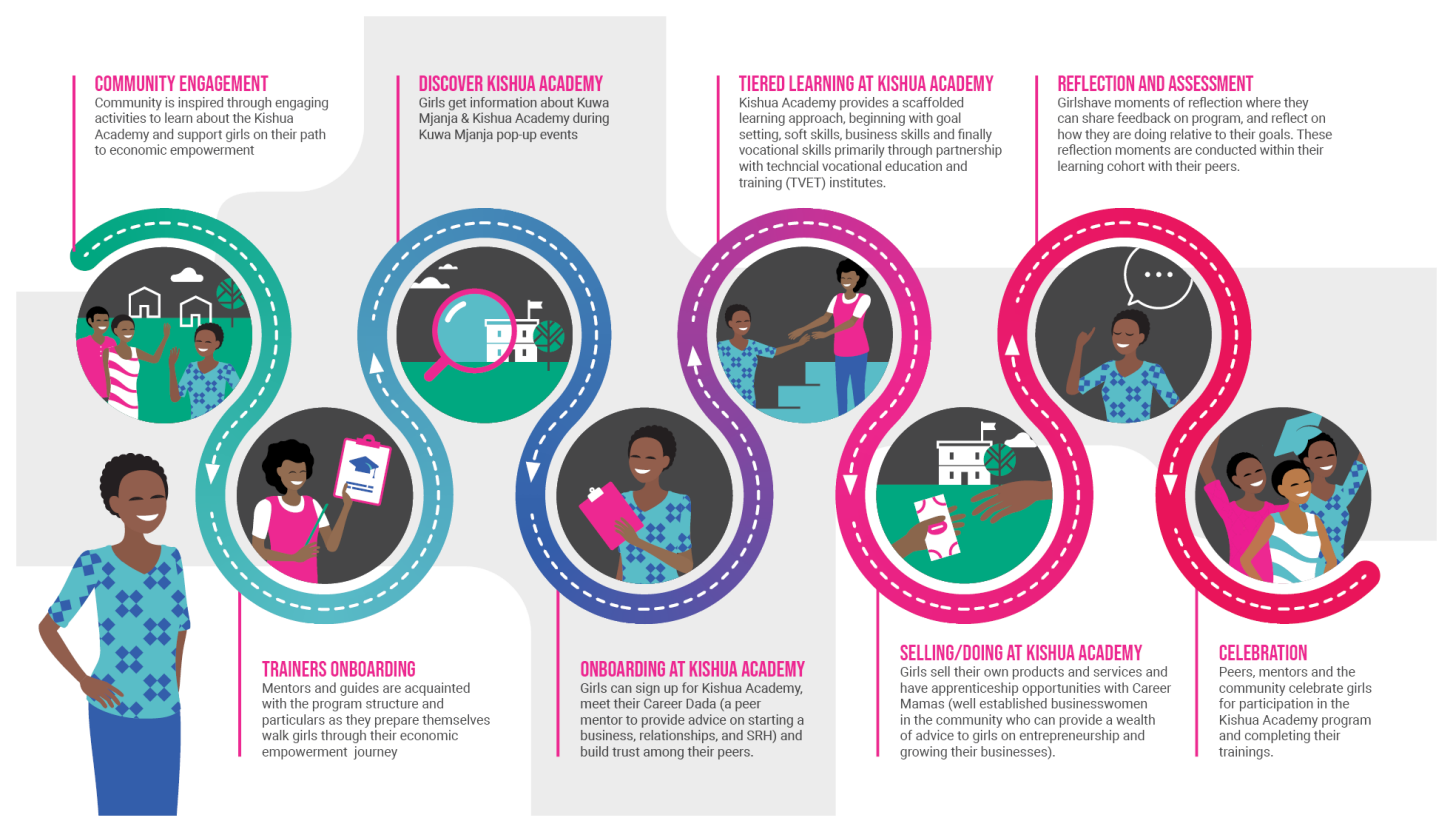
A visual overview of Kishua Academy, the economic empowerment adaptation in Tanzania.

**Figure 6.**
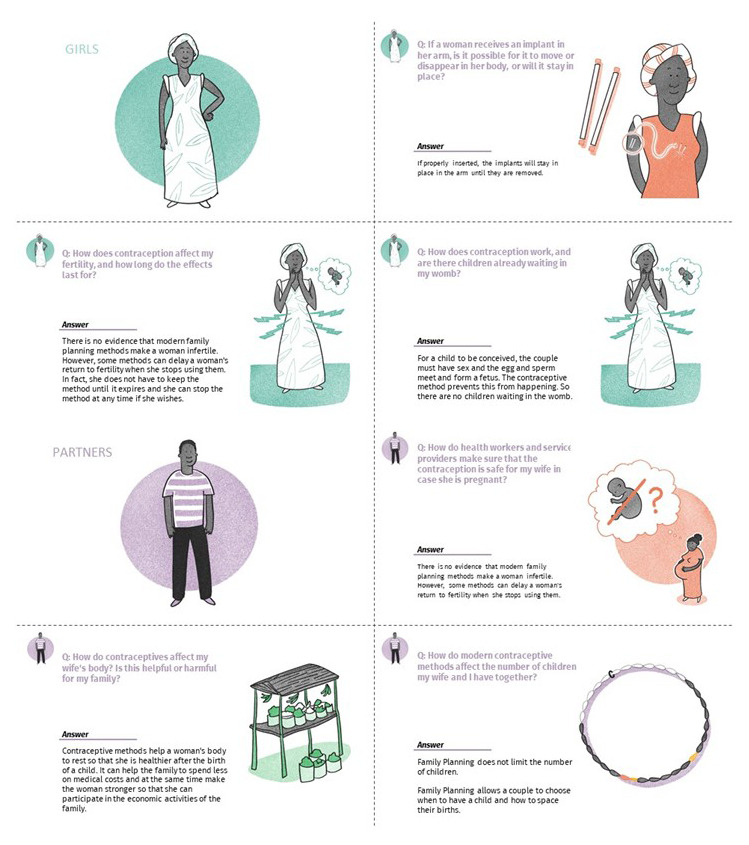
Sample asset, Kishua Academy health facts cards aimed at addressing myths and misconceptions about contraception among girls and their male partners.

In Tanzania, the economic empowerment program **, Kishua Academy**, provides a similar scaffolded skills-building experience as in Nigeria but incorporates additional elements of peer mentorship and partnership with TVET institutes ( Figure 5, Figure 6). Girls are recruited through the SRH intervention, Kuwa Mjanja, which implements community-based contraceptive service delivery events geared towards adolescents. Girls sign up for the Kishua Academy, are introduced to a cohort of their peers with whom they will be receiving skills training, and meet their ‘Career Dada,’ **a peer mentor** with whom they can build trust and have open discussions about their business goals, relationships, and SRH needs. They progress through a series of **soft and business skills sessions** and then are invited to join a specific course at a local TVET institute. Their engagement during and after these **vocational skills training** sessions includes opportunities to **apprentice** with ‘Career Mamas’ who are experienced female entrepreneurs within the community who can mentor and support girls to progress. Girls who opted to learn skills which were predominantly dominated by men in the community were sometimes placed with a ‘Career Baba’, an experienced businessman in the community who could mentor them in their chosen skill. After completing their courses, graduating cohorts **transition to local government oversight** which supports them (through Youth Development Officers / Community Development Officers) to apply for loans to grow their businesses through the local government-run youth development funding program. There is a moment post-graduation for the **community to gather to celebrate girls** for their accomplishment and participation in the program.

In Nigeria and Ethiopia, work moved forward post-design into pilot, allowing for additional refinement, while work in Tanzania ended after live prototyping and no additional refinement was conducted.

## Using evidence during design phrases

Across the design phases, we used the evidence base to inform and contextualize our design process and findings. In doing so we discovered nuance in how what we know from the evidence base corresponded with the insights we were drawing directly from our program users. We have described three overarching categories which depict how we used the existing evidence and new user insights to strengthen our design process. Though we describe these categories separately, in practice there was often overlap.

**1. The evidence base allowed us to expedite finding a solution that worked for our users**

We mined existing systematic reviews to identify promising practices for improving outcomes for adolescent girls around economic empowerment, self-efficacy, and health knowledge ^12, 14, 25– 29^. We prioritized practices that had specific evidence with adolescent populations to build prototypes that could be trialed within our geographies. In the early phases, we intentionally designed inquiry tools and rough prototypes to validate key elements of the evidence base. By speaking directly with adolescent girls and their influencers, we could confirm that they felt these elements were important and obtain additional details on how girls wanted to experience them in practice ( Table 2). This made the process of finding something which worked for our users faster and more efficient. We encountered some limitations in that adolescent girl-focused economic empowerment programming is not traditionally well evaluated, though this is changing with increased investment in this space.

**Table 2.** Validation of insights from evidence with user insights during the economic empowerment design process.

Evidence-based Insights	Validation from User Insights
Adolescents are just beginning to form a deeper sense of who they are and want to be. Developing their skills and capacity is only effective in supporting them to progress towards positive outcomes when support is also provided to help them **establish goals** ** and aspirations and chart a pathway** ** toward achieving those goals** ^31– 34^.	Many if not all the adolescent girls we spoke with were ambitious and eager to make money. Many of them had a specific goal that they stated that they were working towards. At the same time, these goals lacked specificity and girls needed additional support to articulate what exactly they wanted and how they hoped to get there. *“We’ll buy the car when we move to the city. I don’t think we’ll have a problem saving* * enough to buy the car with just our income from agriculture.”* – Married girl, Ethiopia *“I have never written down my goals before because I don’t think I need to, now I have and* * this will guide me in achieving my purposed goal” –* Unmarried girl, Ogun State, Nigeria
Effective programs provide a complementary mix of **vocational, life, and business** ** skills** ^12^.	Girls who had participated in vocational training in the past without a corresponding engagement in soft or business skills found themselves unable to manage their businesses effectively. We routinely saw a lack of key skills required for economic advancement, both in vocational skills training, but also in foundational business skills, such as budgeting and negotiation. *“I have been spending a lot of money and see less profit, so knowing how to manage* * money can help my business and my confidence.”* – Unmarried girl, Tanzania *“Before I had no idea on budgeting and savings but with my participation in the MMA* * program, I ensure all my expenses are budgeted for, I do take my income and minus all my* * expenses from it, this will give me an idea on how much I gain. At the end of my purchase,* * I save the remaining amount. This knowledge I acquired on budgeting and savings has * *helped me to save in group with other girls like myself.”* – Married girl, Kaduna state, Nigeria
There is consensus on the importance of life skills for adolescent girls’ empowerment. Two life skills repeatedly mentioned in the literature as being essential to positive outcomes are **decision-making and** ** negotiation / communication** ^35– 37^.	Adolescent girls struggled to identify which soft skills they needed, as they tended to be more focused on specific hard skills required to jump start income generation or savings. Identifying the types of soft skills needed for advancement is complicated for young people given the rapidly changing world. While they may perceive the gap between their current skills and where they want to be, they may not be able to identify what is required to bridge that gap. In our programming, we adhered to the evidence base and sought to provide effective opportunities to strengthen decision-making and negotiation/ communication skills.
Adolescent girls’ **key influencers provide** ** a key gatekeeping function** to their participation in economic activities ^38, 39^.	Across all geographies, there was a clear need to engage girls’ influencers to increase support for their participation in economic activities. However, this insight came out most strongly in our geographies working specifically with married adolescent girls who experience unique social, religious, and cultural restrictions that constrain their agency and aspirations. *“She can spend her cheese (money) on something small buts if it is something big she need* * to get her husband consent”…“Our husbands don’t allow us stay and sell in shops to protect* *our integrity and image in the eyes of the people.”* – Married girl, Kaduna state, Nigeria *“His [my husband's] support is essential, even if I get money from my parents and buy* * cattle, he might sell them.”* – Married girl, Amhara region, Ethiopia *“My father needs to support me through this journey so that he can be proud of me* * when I utilize the photography skills, I learn to take pictures of all the activities during my* * graduation ceremony.”* – Unmarried girl, Ogun State. Nigeria
Group-based programming for girls frequently uses **mentors to build girls’** ** assets** – for example the relationships, skills, and knowledge she needs to identify and pursue her aspirations. The role of these mentors can vary, but they are a vital part of effective programming for girls ^40^.	There was a clear preference for mentor-led skills training and demonstration over self- guided learning. Through our insights we were also able to dig deeper into the type of profile girls valued in a mentor. Girls saw value both in mentorship from older women who had more business experience and younger ‘near peers’ who they could open up to and who shared similar experiences. *“There should be a mentor who I can always consult through the journey, not only a timed * *program or class.”* – Girl, Tanzania *“I would need support every 3 to 6 months to check back and give me feedback. The first* * time should be in person, then subsequently through SMS or phone calls.” –* Unmarried girl, Ogun state, Nigeria *“I would prefer a mentor to be in charge of our savings collective and also be present* * during savings group meetings.” –* Unmarried girl, Ogun State, Nigeria.

**Figure 7.**
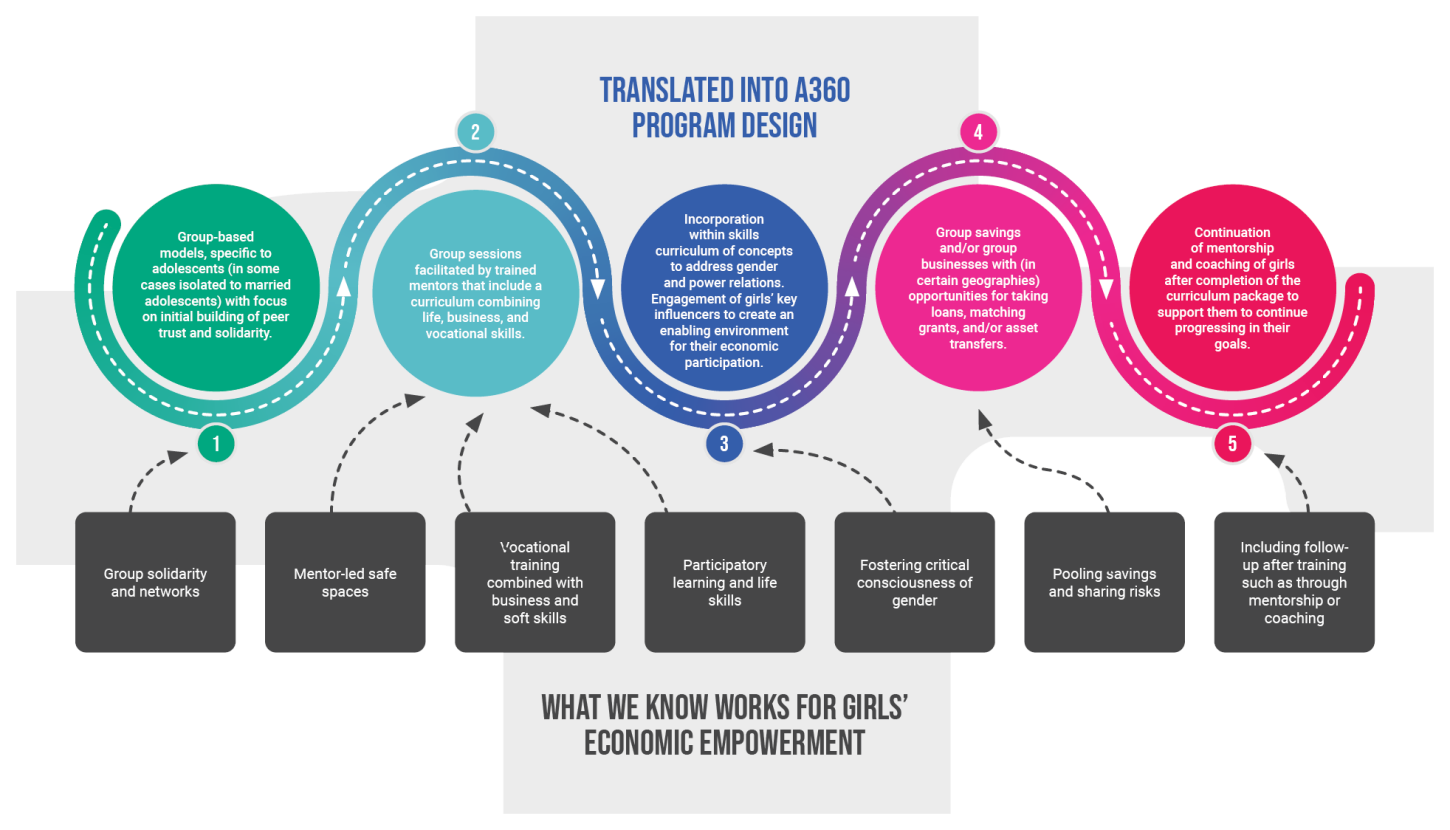
Translation of evidence-based practice into A360 economic empowerment program design.

In general, we understood the practices that have the greatest established efficacy, with some limitations acknowledged, to be a) group-based with opportunities to build solidarity and peer trust; b) mentor-led, particularly by mentors who understand the community context; c) combining vocational training, and business and soft skills development; d) introducing concepts around gender and power to shift girls’ critical consciousness; and e) inclusive of follow up after the initial training, such as through mentorship or coaching ^30^. These components are often most effective when implemented in combination. Additionally, in some contexts we prioritized savings groups, given that they are linked to positive impacts on poverty reduction and can serve as a practical platform to implement the promising practices described above. Despite differences in the final program design across geographies due to varying contextual factors, the resulting programs retained most of these core evidence-based program components ( Figure 7).

Mining existing evidence-based practice provided value not just in insight gathering and initial prototype building, but also moving into the latter design phases (additional rounds of prototype testing and live prototyping) when we needed to build out detailed systems maps. When the design team identified a barrier that needed to be overcome to promote program effectiveness, we first consulted the evidence base to understand if there were existing practices that could be adopted to fill gaps in the proposed program design or strategy.

For example, in line with the broader project objectives to promote sustainability for our program interventions, we wanted to explore pathways to sustainable scale. We examined the evidence base on scaling economic empowerment programs for AGYW and identified key lessons. We learned that good practice required a locally grounded approach with more than one scale pathway, responding to local contexts and generating local ownership ^41^. We recognized that, unlike our SRH interventions, there was not likely to be one partner or government ministry that would have the capacity to wholesale adopt these designs given the cross-sectoral nature of these interventions. Additionally, there are limited resources within ministries to take up these solutions as they are necessarily holistic and need to be delivered with integrity, making them time-intensive and requiring collaboration across multiple stakeholders. This informed our landscaping of potential pathways to scale during live prototyping and helped us focus on understanding which actors were best placed for what during the scale-up process.

As another example, in live prototyping in Ethiopia we noted that though girls showed interest in participating in savings and loans groups, they felt limited by their small savings pool, which constrained the amount of the loans available. This was affecting program attendance and meant girls were missing out on critical soft-skills training sessions. Working with BRAC, we learned about ways other organizations had managed similar challenges. After exploring the options and weighing them against our program priorities, we decided to institute group-level matching grants which groups were eligible to receive contingent on consistent savings and attendance. This was based on an approach successfully implemented by a BRAC partner in Rwanda ^42^.

**2. At times there was a disconnect between what we knew worked in the evidence base and what our users said they wanted.**

Not all attempts to test existing promising practices with our users elicited positive reactions. This is to be expected – adolescent populations are not homogenous, and resonance of these program components is likely to vary across geographic contexts and specific adolescent sub-populations (older vs. younger adolescents, married vs. unmarried, etc.). However, a challenge in HCD can be that users may prioritize what is known or comfortable. If insight gathering and prototyping are not done well, this can lead to users reporting a preference for, at best, the status quo, and worst, harmful traditional practices, as they are the norm. In this instance, having the evidence-base in mind encouraged us to persist in pursuing promising practices and continue learning to understand the barriers that underlay resistance to what the evidence says works. In doing so, we better understood how to effectively apply the evidence base ( Table 3).

**Table 3.** Examples of resolving discordance between the evidence and user insights during economic empowerment design process.

Evidence Insight	User Insight	Final Validated Insight and Tested Solution
**Group-based programming** is shown to be promising for outcomes related to employment, self-efficacy, gender attitudes, and SRH/HIV knowledge ^12^. However, gaps exist in the evidence around group cohesion and trust.	In many instances, particularly for populations of unmarried adolescents, mistrust in girls within peer groups is a common occurrence and is exacerbated when and if financial resources are involved. In the insight gathering phase we found that particularly with populations of unmarried girls they were enthusiastic about learning in groups, but very skeptical about saving or running businesses together. *“The group brand [prototype] does not work because truth is* * hard to find among friends, therefore there will be no trust.”* – Unmarried girl, Ogun state, Nigeria “ *It’s better [to be in a group] so we can all learn from each* * other. If it’s only one person, it won’t be easy and interesting.”* – Unmarried girl, Ogun state, Nigeria *“I want to learn through the group because unity is power.”* – Unmarried girl, Tanzania	In subsequent design phases we continued testing group-based programming and observed to understand the resistance. We assumed that the groups we tested would be focused on learning and that savings and business pursuits would be individual. We made efforts to create cohesiveness and trust in these groups as part of best practice. We found that as trust was built girls saw the potential of shared risk / gain and began pursuing group-based savings and business opportunities on their own. It became clear from this additional testing that girls’ skepticism arose from their lack of exposure to these types of collective ventures rather than their fundamental opposition to them. When they were in an environment with structure that facilitated trust, this skepticism was overcome. *"I would like to do [a savings and rental collective] if the people involved are trustworthy and considerate."* – Girl, Ogun state, Nigeria *"My daughters can do [a savings and rental collective] if they trust themselves. If the trust is there and* * profit is being made, the profits are shared and can be used for other things."* – Mother of girl, Ogun state, Nigeria Our final intervention designs relied on mentor-led, group-based skills classes and incorporated content around building group trust and support in the initial curriculum sessions. We presented girls with the options to pursue individual or collective business ventures but did prioritize group- based savings models (particularly in Ethiopia where this is the core of the final program design).
Programs that support girls with **vocational training** **opportunities should make** ** sure that the skills offered ** **match market needs** ^43^. This can often be facilitated through market assessment, prioritizing youth-led or community-led approaches ^44, 45^. The vocational skills that often show the most promise in graduating girls from small to medium enterprises include those traditionally male-dominated vocations that predominate TVET in most countries ^46^.	Girls gravitated towards learning and practicing traditionally female-dominated skills, often because of the lack of alternative examples within their communities. They were reluctant to learn vocational skills that they weren’t familiar with, as they were concerned about who would patronize this type of business. Additionally, these traditionally female- dominated skills often translated into ‘quicker’ money for girls that could be used immediately, contrasted with the long-term time horizon necessary to learn more complex non-traditional vocational skills (such as electronics or solar panel installation). *“Skills loved to learn by us: soap making, make up, bead making,* * tailoring, tie and dye making, ofi making, clothes knitting, gele* * tying, playing instruments and catering.”* – Unmarried girl, Ogun state, Nigeria *“My parents are farmers, I don’t feel like I need to go to* * [vocational training]. I’m comfortable doing what my parents are* * doing.”* – Girl, Tanzania	In further exploration, it was possible to prompt girls’ interest in exploring new skills through critical consciousness activities such as asking girls why certain tasks were for men or women. Some girls even changed the skills they selected as most interesting as a result, after asking whether “girls could do a new or different skill,” such as learning to become an electrician. Yet new skills still felt like a risk for girls who were reliant on earning quick money to support themselves or who had limited family support. *“I feel comfortable with the traditional skills because I can learn them at any time and there would always* * be someone to teach me. I feel discouraged about the non-traditional skills because the materials are not * *readily available.”* – Married girl, Kaduna state, Nigeria In the final intervention design for our geographies that included vocational skills training, we elected to give girls the opportunity to learn more than one vocational skill. This allowed girls to pursue the ‘quick wins’ that often came through the learning and application of more traditional skills while also taking advantage of the potential that non-traditional skills could bring in opening greater market opportunities. Yet, the reality of entrenched gender norms still restricted the skills girls chose to learn, with only a small subset of girls choosing what they perceived as the ‘riskier’ non-traditional skills.
Evidence **emphasizes the** ** importance of life skills for** ** improving a range of outcomes** ** for adolescent girls**, including psychosocial and mental health, SRH, social relationships, and economic assets and opportunities ^24, 29, 47– 55^. Life skills can also be linked to improved self-efficacy and agency among adolescent girls ^48, 56^.	Girls conveyed some reluctance to attend soft skills curriculum sessions. In some instances, girls had already failed school and were uncomfortable in classroom-like settings. In others, they were unclear on the value of life-skills relative to more obvious benefits of saving money or learning a vocational skill. This impacted program attendance in unexpected ways. For instance, in Ethiopia, girls would miss a session, but still send money with a friend to contribute to that week’s saving. *“I started feeling a headache because [these skills sessions] made* * me think too much.”* – Married girl, Kaduna state, Nigeria	We hypothesized that if we could find a way to get girls beyond that initial reluctance to attend these sessions, that they would be able to appreciate the value of the skills. We adapted our strategy for the classes to be a low-literacy, participatory approach based on BRAC’s ELA program. In addition, we made design choices that promoted attendance at these sessions (i.e. linking eligibility for matching grants to session attendance in Ethiopia). Ultimately, we did see that girls had a positive experience and valued these sessions after they were prompted to attend. Our testing also reinforced the value of quality delivery of the content by a well-trained mentor. Mentors that were not well trained tended to default to lecture-based, non-participatory approaches that discouraged and disincentivized girls from engaging.

**3. New insights allowed us to build a greater understanding of our users’ lived experiences where there were existing gaps in the evidence base**

The evidence on promising practices for girls’ economic empowerment, though growing, is not exhaustive. In conducting our insight-gathering phase, we were able to uncover additional detail on the lived experiences of our program users that did not feature prominently in the evidence base. While the overall themes of the insights drawn from the design process are not new, the context-specific nuances were important for us in program development and can be valuable contributions to the existing body of evidence.

***Increasing resilience through a diversity of skills***

In the evidence base we saw that most programs focused on technical skills paired with some business or soft skills. Yet, our research demonstrated an enormous appetite by girls to learn a diverse set of skills that could provide them with a competitive advantage. Girls did not want one-dimensional training; they were eager for continuous upskilling that would allow them to weather shifts in the market and other situational factors, such as the impacts of climate shocks or conflict. They were eager to learn skills that could be layered together for greater profit, such as hairdressing and make-up or poultry rearing and fish farming. Best practice and research, in our exploration of the literature, did not address the need for young female entrepreneurs to have a set of skills that could allow them to adapt to seasons and trends to ensure continuous income throughout the year and in a longer-term time horizon. Designing a skills curriculum around this type of ‘resilience’ could also become even more critical as the impacts of climate change are felt more acutely within these communities.

*“I don’t want to learn about soap-making only.”* – Girl, Tanzania
*“If we have a lot of businesses in our group…the clients will have a lot of choice to choose what they want.”* – Girl, Tanzania
*“I want to own my own business in the future*. *I would like to own a hairdressing / mani-pedi business. Make-up will give me the most income…I’ll learn all the skills around make-up, pedicure, and manicure so I can have my own place.” *– Girl, Ogun State, Nigeria (currently working as a make-up artist)

***Navigating business choices and market access for girls in conservative settings***

In certain geographies our design research uncovered greater specificity in the restrictions faced by married adolescent girls in conservative contexts that influence the ways in which they can enter and participate in the market. In northern Nigeria especially, where mixing between the sexes is restricted, girls were often severely limited in options for income-generating activities. In Ethiopia, although there is more interaction between the sexes, the result is much the same, as traditional gender roles mean that girls must be at home to take care of children. Yet, girls demonstrated incredible entrepreneurialism in the face of those restrictions. In Nigeria, girls participated in women-only markets on dedicated days or worked out of their home and relied on husbands or brothers to sell their products. In Ethiopia, girls focused on home-based income-generation activities (such as poultry rearing) or negotiated with extended family members to shift childcare responsibilities. These insights point to how adolescent girls, and women in general, can be successful in income generating activities in conservative settings. Nonetheless, the social practice of sex-based restrictions (e.g. male only markets) has a significant impact on entrepreneurship opportunities and the ways these girls must navigate business. This uncovered a need for further research and best practices on how women can work within these existing restrictions even while systems and norm change are in process.

*“Women run their businesses at home and there is a market just for women (on Thursdays) where they trade and shop.”* – Girl, Kaduna state, Nigeria
*"So far, we don't support each other. I work on the farm, she works at home. Maybe going forward we can help each other." –* Husband of an adolescent girl, Ethiopia,
* “Most women are not into shoe making and in the whole of [LGA name] only two men are into shoe making*… *Yes, my husband would let me do it, but he won’t let me open a shop, so I would have to do it at home.” *– Girl, Kaduna state, Nigeria

***Low trust in institutional support structures***

While we expected to find some frustration or disappointment with prior financial inclusion initiatives that had ended or failed to provide the promised impact, the level of mistrust was greater than we anticipated. We were struck by the broad-based level of mistrust expressed by participants stemming from their interaction across non-governmental, governmental, and formal financial institutions. Users pointed to the failures of past NGO projects to promote lasting outcomes. Mistrust of government-run programs often stemmed from corruption, burdensome bureaucracy, or poor management that limited the funds available for community services. Some users, however, reported greater trust in government loan programs, as rates were generally considered favorable. This contrasts sharply with opinions about microfinance institutions. Across geographies, participants shared negative experiences about high interest rates that made loans difficult, if not impossible, to repay.

These negative perceptions raise questions about how NGOs should be interacting with institutional structures to address persistent gaps and promote sustained change. Leveraging existing systems generally presents the greatest avenue for sustainability. However, given existing mistrust and structural deficiencies we question the feasibility of this without significant structural change. Our insights point to a need for the organizations promoting economic empowerment, especially for marginalized groups, to move beyond understanding these barriers to intentionally designing and implementing programming that pursues strengthening of financial institutions in a way which is sustainable and promotes community trust.

*"LAPO [microfinance institution] are killers."* – Mother of adolescent girl, Ogun state, Nigeria
*"I would not be interested [in government loans]. I do not have a reason, but the government are not useful, fair, and I would not want to use them."* – Girl, Ogun state, Nigeria

***Variations in desired and required dosage for achieving economic empowerment***

Across documented skill-building programming for adolescent girls, the topics covered, and time dedicated, differed, from a week to over a year long. The research on the appropriate dosage needed to achieve positive outcomes is limited. This leaves organizations to make their best guess about an appropriate timeline and to let budgetary limitations drive decision-making. Dosage is complicated by disparate needs across sub-populations of adolescents. We saw this across our own geographies in how insights led to differences in dosage – in northern Nigeria, our program provided 6–8 hours of SRH and soft skills sessions and 15–20 hours of economic empowerment training including soft and hard skills over the course of 12 weeks. While in Ethiopia, participants met for one and a half hours per week over the course of approximately 30 weeks. We struggled to balance the time girls needed to absorb skills and new information against the other demands on their time. Married girls were busy managing their household and caring for children. Younger unmarried girls were attending school and had a full load of chores at home, and older unmarried girls might already be involved in a basic income generation activity. In cases where girls were unable to leave home due to pregnancy or childbirth, we provided make up sessions to participants. Despite initial resistance mentioned previously to participate in soft skills training, attendance across geographies for these skills sessions was high, demonstrating that localized solutions with attention to the varying needs of different target populations are critical. These insights also point to the value in providing alternative learning pathways for individuals when they encounter challenges in attending regular group meetings for periods of time.

***Leveraging joint goal setting and crafting long term engagement plans for girls’ influencers***

While the evidence base is clear on the importance of engaging married girls’ husbands to support their participation in economic empowerment activities, there are fewer evidence-based strategies on how to do so effectively. In Ethiopia, where our SRH approach already targets couples, we used an extended goal-setting session to try and build this support. During the session, which included a facilitated conversation between the couple, they identified their long-term goals and how they would each contribute. Using a model couple, we demonstrated how goals could be reached sooner if both the husband and the wife participated in income generation. We took care to note the non-income-generating activities often falling on women (such as childcare or food preparation) that also contribute to the success of the household. At the end of the session, husbands were introduced to the economic empowerment program and asked to support their wife’s attendance. Both husbands and wives responded positively to these sessions, suggesting the value of harnessing shared goal setting and in making explicit the benefits of multiple earners. While this was a positive start, additional contact with husbands was needed. Some husbands required multiple visits from program facilitators to reinforce the importance of their wife’s attendance at weekly association meetings. In addition, it is essential to be clear what participants would and would not receive for participation. Some husbands expected that their wives would receive a cash transfer for participation and grew frustrated when that was not the case. Again, this required additional communication from program facilitators with husbands to resolve. On the other hand, we also saw some husbands who were very supportive of their wife’s participation, as demonstrated through attending sessions to contribute weekly savings if she was unable to attend.

## Lessons and recommendations

This open letter describes the complexity we encountered while trying to design with responsiveness to the existing evidence and to users’ desires and needs. A challenge we faced was that the evidence for economic empowerment among adolescent girls was not easily translatable to program design. First, the volume of evidence around economic empowerment programs for adolescents often seemed overwhelming. Second, the evidence base has significant gaps in research and practice around girls’ economic empowerment, particularly marginalized communities ^12^. The translation of evidence was exacerbated by the differences between the macro-economic contexts in which we are implementing. For example, a lack of vocational training centers or high levels of unemployment limit the opportunities available and what proven interventions may even be plausible. Additionally, as an organization focused on improving health and not economic outcomes, we were at a disadvantage. We know that girls need – and ask for – multi-sectoral solutions. In the absence of well-established collaboration across sectors, this may require organizations to pursue design and implementation of programs outside their existing wheelhouse.

We found that the HCD process helped us to translate the evidence and make it more easily usable to program designers. For example, as noted above, the insight gathering and prototyping processes allowed us to validate existing insights from the evidence base and hear more from girls about what they wanted to see in practice, for instance the need to help girls get more specific with their goal setting or help them to see the value of soft skills classes. The HCD process also allowed us to fill in gaps in the existing evidence base and supported us to adopt user-generated solutions, such as the need to offer multiple vocational skills to help girls stay competitive.

Our economic strengthening technical partner also provided invaluable feedback and allowed our teams to rapidly expand their technical capacity in this new program area. We adopted two approaches to reduce the burden on teams to absorb too much information at once. The first was that the economic empowerment partner took on the role of synthesizing large volumes of information. Rather than share journal articles or large reviews, the partner developed presentations on key topics that lasted approximately an hour and allowed time for discussion. The second was to have an “on-demand” support mechanism as questions or challenges arose. Given the vast amount of information available, much of which might be irrelevant depending on the program model chosen, it did not feel efficient to try and proactively understand evidence around potential future challenges. It was important to provide design team members, especially those based in implementation geographies, the right information at the right time. In this way having a technical assistance approach which was demand-based allowed us to request detailed information on a specific challenge as it arose and focus our energies on understanding and applying the evidence. Nonetheless, we believe it would be better to have technical expertise fully embedded within the design team to support in the identification of gap areas as they arose, as a less experienced implementor may not always be able to recognize known gaps or program weaknesses.

Though we instituted many ‘layers’ of technical support to promote translation and application of the evidence, we know that despite our best intentions there were pieces of evidence that we missed the opportunity to apply because we didn’t have the bandwidth to digest such a substantive body of knowledge. The volume of information to absorb almost guarantees that organizations will miss out on the opportunity to apply certain evidence-based practices. We encourage researchers to consider how to make evidence more easily digestible to practitioners and for the whole community of practice to work together to identify what questions need to be asked to effectively operationalize evidence in a local context.

## Data Availability

The quotes and synthesis of data from design included within this article are intended to provide examples to substantiate our learning and experience from the design process described in this Open Letter. There are therefore currently no raw data associated with this Open Letter. However, a synthesis of the data and learning generated during design will be published externally at the end of 2023.
