# Supplementary material for: Balancing evidence-informed and user-responsive design: Experience with human-centered design to generate layered economic empowerment and SRH programming in Tanzania, Ethiopia, and Nigeria
Source: Gates Open Res. 2023 Jul 4;7:106. [Version 1] doi: 10.12688/gatesopenres.14724.1 (PMC10547119; doi:10.12688/gatesopenres.14724.1)
Supplement: Peer review history

# Reviewer report: Reviewer response for version 1

**DOI:** 10.21956/gatesopenres.16050.r34588

**Published:** 2023-09-12

**Authors:** Anne LaFond, Muthoni Wachira

**Affiliations:** 1 JSI, Arlington, USA

The letter explains clearly the importance of layering ASRH programs with economic empowerment opportunities. It also communicates well the complexity associated with this kind of programming and the challenges of applying both evidence-based practices and user-responsive strategies. The paper provides valuable information not only for ASRH and health program funders, designers and implementers, but also programs in other sectors that seek to address the intersectionality of social outcomes and economic opportunity and security. It delves deeper than most papers into specific and practical lessons of integrated program designs and the use of two best practices: being evidence-informed and generating and applying insights on program users’ lived experiences, especially among vulnerable and under-represented populations. The experiences/insights shared from this program will benefit ASRH programs seeking better and more sustained ASRH outcomes. The call for improving approaches to synthesizing evidence and making it accessible (and therefore useful) to program designers should be noted.

 An advantage of this paper beyond providing insights into programs that layer economic empowerment interventions into SRH is its focus on how the program design process took place. This paper offers practical insights into intervention design strategies that seek to optimize both human-centered and evidence-driven approaches without backing away from the challenge of working this way. Consultation with program users may be a standard practice. However, HCD introduces ways of working with people (youth in this case) that generates insights that may not be well reflected in published evidence. In the case of the four programs discussed, these user insights helped to tailor and refine evidence-driven health programs and strengthen intervention design. The layering of evidence, along with collaboration with economic empowerment experts, helped program designers find the right fit between interventions and a variety of youth program users across different countries, age groups, and life stages.   

 Tables 2 and 3 are particularly useful in illustrating how evidence and user insights can be complementary in program design. Where evidence provides a range of tested solutions (e.g., the core components that made up each intervention), HCD helps to validate their relevance in specific program settings and populations, fill evidence gaps and streamline the prioritization of possible solutions. Where user insights did not line up fully with the published evidence, the HCD processes - user insights research and prototyping - helped to tailor interventions to the needs and desires of different users (e.g., younger and older adolescents; married, unmarried). The discord between evidence-based approaches and user insights was addressed through iteration and adaptation in the application of HCD.

 To further the application of this learning, it would be helpful if the authors could translate their learnings more explicitly for practitioners who are designing HCD-led interventions.

**Suggested minor revisions:** 

1. While the focus on being evidence-informed is clear in the paper, the authors could explain more clearly how the design process was “gender-intentional.”
2. It would help to reflect on the effectiveness of the balanced approach as well as its complexity. If there is any available evidence on the outcomes of any of the programs presented, it would be useful to mention it with the caveat that economic empowerment programs can take some time to produce results.
3. Provide a brief explanation of what you mean by age-appropriate economic empowerment. Questions may arise around the a) legal age for employment in the three countries and b) legal and ethical considerations around work for 15 and 16-year-olds. It would be helpful to understand if this influenced project design or success.  
4. Economic empowerment programs are gaining traction in ASRH programing. It would be valuable for this letter to recommend areas for further research to determine the influence of economic empowerment on ASRH programs.
5. P9 Header. There is a typo in ‘ *Using evidence during design phrases’ It should be ‘design phases’.*

Does the article adequately reference differing views and opinions?

Yes

Are all factual statements correct, and are statements and arguments made adequately supported by citations?

Yes

Is the Open Letter written in accessible language?

Yes

Where applicable, are recommendations and next steps explained clearly for others to follow?

Partly

Is the rationale for the Open Letter provided in sufficient detail?

Yes

Reviewer Expertise:

NA

We confirm that we have read this submission and believe that we have an appropriate level of expertise to confirm that it is of an acceptable scientific standard.

# Reviewer report: Reviewer response for version 1

**DOI:** 10.21956/gatesopenres.16050.r34586

**Published:** 2023-08-25

**Authors:** Louise Fox

**Affiliations:** 1 The Brookings Institution, Washington, District of Columbia, USA

This Open Letter describes Gates-funded project experience delivering SRH services to female adolescents in Sub-Saharan Africa (SSA) using gender transformative programming. The grantees used a human-centered design (HCD) process to solicit early feedback from stakeholders (e.g. potential participants, their families, and community leaders) on the participants' perceived needs and ideal modes of service delivery. Service providers incorporated this feedback into project design.

 Key findings included:  

- Participants did not initially value SRH services and associated life skills training as much as the providers did based on international evidence. 
- Participants wanted vocational skills training to help with income generation (e.g. hair dressing skills, business skills). They were more willing to participate in other trainings if they also got the vocational skills training.
- Participants mostly sought skills training in traditionally female fields, despite these not being the most lucrative.  Participants chose the "known and comfortable".  
- Engaging with stakeholders (especially the family) at the outset was highly effective. In particular, engaging with husbands on joint goal setting proved effective.

These insights are very valuable for practitioners, and clearly demonstrate the value of the HCD approach. While the cheapest design for an SRH project could be to simply provide the SRH services (which, if used, tend to have a high rate of return), if the target population does not demand these services and does not value them relative to vocational training, the cheapest design will be the least cost-effective. These findings help justify the use of more expensive but multi-faceted program design. They may also explain why many NGO- or donor-led programs for adolescent females do not scale into the public sector, where implementation silos dominate.

 The practitioners' experience also highlighted the limits imposed by existing gender norms on the use of a gender transformative service provision model. The Letter also highlighted a known but undervalued result: that the success of life skills training is highly dependent on the quality of the mentor and the pedagogical methods.

 Overall, the Letter is well written and presents the programming and results in clear writing and very helpful graphics. However, the introduction was less helpful and should be revised. A stronger introduction would include:

- Statistics on female adolescents in SSA, not global statistics which are mostly meaningless in this context.
- A summary paragraph on what we know (evidence) about why adolescents in SSA have such a high rate of unmet contraception need compared with all women of reproductive age. This material would (i) set the scene for the program design, and (ii) help explain the findings. Ideally this discussion would go beyond the vague "discriminatory gender norms and patriarchal structures" since presumably these constrain all women, not just adolescents.

Finally, although this Letter focused on practice, not causal evidence, some statistics on outcomes (including attrition, which presumably was lower owing to the HCD design approach) would be helpful.

Does the article adequately reference differing views and opinions?

Partly

Are all factual statements correct, and are statements and arguments made adequately supported by citations?

Yes

Is the Open Letter written in accessible language?

Yes

Where applicable, are recommendations and next steps explained clearly for others to follow?

Yes

Is the rationale for the Open Letter provided in sufficient detail?

Yes

Reviewer Expertise:

Youth Employment in Sub-Saharan Africa.

I confirm that I have read this submission and believe that I have an appropriate level of expertise to confirm that it is of an acceptable scientific standard.

# Reviewer report: Reviewer response for version 1

**DOI:** 10.21956/gatesopenres.16050.r34581

**Published:** 2023-08-15

**Authors:** Rajat Khosla

**Affiliations:** 1 United Nations University, Kuala Lumpur, Malaysia

This is a very well researched and timely article and I congratulate the authors. Using HCD process to layer complementary economic empowerment components to existing SRH interventions is a very useful way to ensure sustainability of interventions and prevent high turnover of clients. 

 The manuscript would benefit further from additional insights/details on the following elements: 

 a) how does the layering of economic empowerment components to SRH interventions help navigate (positively or otherwise) the family/community dynamics and power relations associated with certain SRH interventions eg family planning; CSE etc; 

 b) do the retention rates improve for SRH components after the economic interventions stop; 

 c) Is there a "compromise" element to be factored in, i.e. do SRH interventions have to lowered in ambition for the package of SRH/economic empowerment to be accepted by clients and communities. For instance, does it preclude addressing certain SRH components over the others, e.g safe abortion; sexuality; 

 d) Did the study look into dimensions which should be factored in to ensure scale, i.e. how to you move this from boutique level to scale. 

 These are just some additional issues which would be useful for authors to address as they finalise the manuscript and develop next steps.

Does the article adequately reference differing views and opinions?

Yes

Are all factual statements correct, and are statements and arguments made adequately supported by citations?

Yes

Is the Open Letter written in accessible language?

Yes

Where applicable, are recommendations and next steps explained clearly for others to follow?

Partly

Is the rationale for the Open Letter provided in sufficient detail?

Yes

Reviewer Expertise:

Gender equality, health eequity, SRH

I confirm that I have read this submission and believe that I have an appropriate level of expertise to confirm that it is of an acceptable scientific standard, however I have significant reservations, as outlined above.
